# Secondary Sex Ratio among Women Exposed to Diethylstilbestrol *in Utero*

**DOI:** 10.1289/ehp.10246

**Published:** 2007-06-28

**Authors:** Lauren A. Wise, Julie R. Palmer, Elizabeth E. Hatch, Rebecca Troisi, Linda Titus-Ernstoff, Arthur L. Herbst, Raymond Kaufman, Kenneth L. Noller, Robert N. Hoover

**Affiliations:** 1 Slone Epidemiology Center, Boston University, Boston, Massachusetts, USA; 2 Department of Epidemiology, Boston University School of Public Health, Boston, Massachusetts, USA; 3 Norris Cotton Cancer Center, Dartmouth Hitchcock Medical Center, Lebanon, New Hampshire, USA; 4 Division of Cancer Epidemiology and Genetics, National Cancer Institute, National Institutes of Health, Department of Health and Human Services, Bethesda, Maryland, USA; 5 Department of Obstetrics and Gynecology, University of Chicago, Chicago, Illinois, USA; 6 Department of Obstetrics and Gynecology, Baylor College of Medicine, Houston, Texas, USA; 7 Department of Obstetrics and Gynecology, New England Medical Center, Boston, Massachusetts, USA

**Keywords:** diethylstilbestrol, estrogens, endocrine-disrupting chemicals, females, sex ratios

## Abstract

**Background:**

Diethylstilbestrol (DES), a synthetic estrogen widely prescribed to pregnant women during the mid-1900s, is a potent endocrine disruptor. Previous studies have suggested an association between endocrine-disrupting compounds and secondary sex ratio.

**Methods:**

Data were provided by women participating in the National Cancer Institute (NCI) DES Combined Cohort Study. We used generalized estimating equations to estimate odds ratios (ORs) and 95% confidence intervals (CIs) for the relation of *in utero* DES exposure to sex ratio (proportion of male births). Models were adjusted for maternal age, child’s birth year, parity, and cohort, and accounted for clustering among women with multiple pregnancies.

**Results:**

The OR for having a male birth comparing DES-exposed to unexposed women was 1.05 (95% CI, 0.95–1.17). For exposed women with complete data on cumulative DES dose and timing (33%), those first exposed to DES earlier in gestation and to higher doses had the highest odds of having a male birth. The ORs were 0.91 (95% C, 0.65–1.27) for first exposure at ≥ 13 weeks gestation to < 5 g DES; 0.95 (95% CI, 0.71–1.27) for first exposure at ≥ 13 weeks to ≥ 5 g; 1.16 (95% CI, 0.96–1.41) for first exposure at < 13 weeks to < 5 g; and 1.24 (95% CI, 1.04–1.48) for first exposure at < 13 weeks to ≥ 5 g compared with no exposure. Results did not vary appreciably by maternal age, parity, cohort, or infertility history.

**Conclusions:**

Overall, no association was observed between *in utero* DES exposure and secondary sex ratio, but a significant increase in the proportion of male births was found among women first exposed to DES earlier in gestation and to a higher cumulative dose.

Diethylstilbestrol (DES) is a synthetic estrogen that was prescribed to > 2 million pregnant women in the mid-1900s. In later years, it was discovered to be associated with the occurrence of vaginal clear cell adenocarcinoma (Herbst et al. 1971), anatomic abnormalities of the reproductive tract (Stillman 1982), and poor reproductive outcomes in daughters (Beral and Colwell 1981; Kaufman et al. 2000; Palmer et al. 2001). DES exposure *in utero* may also exert long-term effects on female endocrine function, possibly leading to permanent dysregulation of the hypothalamic–pituitary–ovarian axis and alterations in hormone biosynthesis in adult women (Assies 1991; Peress et al. 1982; Wu et al. 1980). Although the effect of DES on plasma sex hormones has not been well studied, elevated levels of serum testosterone (Wu et al. 1980), but not luteinizing hormone (LH) (Peress et al. 1982; Wu et al. 1980), progesterone (Wu et al. 1980), or estrogens (Peress et al. 1982; Wu et al. 1980), have been documented in DES daughters. Levels of follicle-stimulating hormone (FSH) were elevated in DES-exposed women in one study, but no differences were found in LH or the ratio of FSH to LH (Peress et al. 1982); another study found no difference in FSH levels (Wu et al. 1980). Animal data show that *in vitro* production of testosterone, total estrogen, and progesterone by ovarian tissue was significantly greater in female mice exposed prenatally to DES, at all ages studied (Haney et al. 1984).

Secondary sex ratio (proportion of male births)—a prevalence measure that reflects both sex programming at the time of conception and survival until birth—may be influenced by exposure to endocrine disruptors such as DES. In humans, several studies have examined the relation between preconceptual exposure to endocrine-disrupting compounds and secondary sex ratio, but most associations were observed in men and were mixed in direction (James 2006). With respect to maternal exposure, a significant decrease in sex ratio was found in studies of polychlorinated biphenyls (PCBs) (Weisskopf et al. 2003) and mercury (Sakamoto et al. 2001), but the majority of studies have been null, including those that examined maternal exposure to dioxin (Mocarelli et al. 2000; Rogan et al. 1999; Ryan et al. 2002; Yoshimura et al. 2001), PCBs (del rio Gomez et al. 2002; Karmaus et al. 2002; Taylor et al. 2006; Taylor et al. 1989), lead (Jarrell et al. 2006), and dichlorodiphenyltrichloroethane (DDT) (Cocco et al. 2006; Karmaus et al. 2002). Moreover, dose and timing of exposure have been incompletely characterized in many studies, and little is known about the relation of these chemicals to the maternal endogenous hormonal milieu. For instance, PCBs have estrogenic, antiestrogenic, and androgenic properties (Bonefeld-Jorgensen et al. 2001), making the direction and magnitude of their effects difficult to predict. In a small study of preconception maternal PCB concentrations that stratified by hormonal activity of the PCB, Taylor et al. (2006) found that the odds of a male birth were elevated among women exposed to estrogenic but not antiestrogenic PCBs. Although not statistically significant, these results suggest that PCBs have different biologic effects depending on their underlying hormonal activity.

A prevailing hypothesis is that endocrine disruptors such as DES may affect secondary sex ratio through changes in hormonal concentrations around the time of conception (James 1987). In women, high levels of gonadotropins (FSH and LH) and progesterone are hypothesized to change the ratio toward more girls, whereas high testosterone and estrogen levels change the ratio toward more boys (James 1987). Another hypothesis, the “over-ripeness ovopathy” theory (Jongbloet 2004), postulates that sex ratio is influenced by both oocyte maturation and the quality of cervical mucus, with nonoptimal hormonal modulation favoring male-biased progeny. Nonoptimal liquefaction of cervical mucus may facilitate differential migration of sperm, with increased fertilization by Y-bearing sperm because the head, length, perimeter, and area are significantly smaller and the neck and tail are shorter in Y-bearing sperm than in X-bearing sperm (Cui 1997). Because concurrence of both oocyte maturation and cervical mucus liquefaction is modulated by estrogens before the midcycle, any perturbations to the endogenous estrogenic milieu caused by endocrine disruptors may theoretically affect sex ratio (Jongbloet 2004).

To our knowledge, there are no studies of secondary sex ratio in women exposed to DES, either prenatally or preconceptually, and most animal studies of this association are null. Specifically, studies in female mice (Honma et al. 2002; Suzuki et al. 2002), rats (Odum et al. 2002), and Chinese rare minnows (Zhong et al. 2005) have found no association between prenatal DES exposure and secondary sex ratio, whereas studies in rats exposed pre-conceptually to DES had an increased proportion of male offspring (Sharpe et al. 1995).

We evaluated the association between *in utero* DES exposure in women and the secondary sex ratio of their offspring in a large collaborative study of participants with and without documented exposure to DES. Based on previous studies of sex steroid hormone levels in women exposed *in utero* to DES and the possible influence of these hormones on sex ratio (James 1987), we hypothesized that DES-exposed women would have a higher proportion of male offspring than unexposed women.

## Materials and Methods

### Study population

The National Cancer Institute (NCI) DES Combined Cohort Study began in 1992 and includes four individual cohorts of DES-exposed and unexposed women that combine participants from several field centers. The methods of the original studies from which these cohorts were assembled have been described elsewhere (Bibbo et al. 1977; Colton et al. 1993; Dieckmann et al. 1953; Horne and Kundsin 1985; Labarthe et al. 1978). Briefly, participants from three cohorts originally identified for study during the 1950s–1970s—the Diethylstilbestrol Adenosis Project (DESAD; Labarthe et al. 1978), Dieckmann (Bibbo et al. 1977; Dieckmann et al. 1953), and Horne (Horne and Kundsin 1985)—were traced and contacted for follow-up by the NCI. The Dieckmann cohort study was a clinical trial designed to test the efficacy of DES in preventing adverse pregnancy outcomes among women receiving routine prenatal care at the University of Chicago (Chicago, IL). The DESAD and Horne cohorts were derived from clinic populations. A fourth cohort, the Women’s Health Study (WHS), included the female offspring of women who had participated in a 1970s health study of DES-exposed and unexposed mothers. The study protocol was approved by the human subjects committees at the field centers and by the NCI. Women provided informed consent by completing and returning the mailed questionnaires, or by participating in a telephone interview.

From the four cohorts, 7,439 daughters were identified in 1992 as eligible for follow-up. Of these, 84 were deceased, and 804 had refused further contact during the original cohort studies or were untraceable. In 1994, 6,551 study participants (88% of the original surviving cohort) were contacted by mail and were sent a baseline questionnaire eliciting information on reproductive and contraceptive history, lifestyle and behavioral factors, medical conditions, medication use, and health care utilization. Participants were called for a telephone interview if they did not respond to two mailed questionnaires. In 1997, a follow-up questionnaire was sent to update reproductive and medical information. A total of 5,707 (87%) participants responded to the 1994 questionnaire (3,946 exposed and 1,761 unexposed), and 5,579 (85%) responded to the 1997 questionnaire (3,893 exposed and 1,686 unexposed). For the present study, we included women who completed at least the 1994 questionnaire. We then excluded nulliparous women [1,433 (41%) exposed and 481 (27%) unexposed], parous women who did not give information on offspring sex [16 (< 1%) exposed and 5 (< 1%) unexposed], and women who reported multifetal births but no singletons [1 (< 1%) exposed and 0 (< 1%) unexposed].

### Assessment of exposure, outcome, and covariates

DES exposure status was verified in all cohorts by medical record. The completeness of data on DES dose and gestational timing of first exposure varied across the four cohorts. Data on DES dose and timing were carefully documented in the Dieckmann cohort, with participants being exposed to high cumulative doses of DES (median cumulative dose, ~ 12 g) in adherence to the regimen of Smith and Smith (1949). Women in the Horne cohort were generally given high doses of DES, and records of dose and timing were available on almost all women. Exposure in the DESAD cohort was difficult to estimate because of incomplete information from medical records. In this cohort, estimates ranged from a median cumulative dose of approximately 1.5–2.5 g at Baylor College of Medicine (Houston, TX) and the Mayo Clinic (Rochester, MN) to 8.5 g at the Boston Lying In Hospital (Boston, MA) (Labarthe et al. 1978). Data on dose and timing were unavailable in the WHS cohort.

Information on DES dose was available for 36% of exposed women. We used 5 g as a cutpoint for “low” versus “high” dose because the distribution was bimodal, with peaks at about 2 g and 12 g. The cohorts included women from several regions of the United States. As regional DES prescribing practices were similar, we conducted a secondary analysis in which women with missing data on dose were assigned an imputed value based on the median dose for their specific field center. Assigned doses were as follows: Dieckmann, 12.4 g; Horne, 10.4 g; Boston/DESAD, 8.5 g; California/DESAD, 7.9 g; Baylor/DESAD, 2.6 g; Wisconsin/DESAD, 3.2 g; and Mayo/DESAD, 1.5 g.

Gestational age at first DES exposure (in weeks), available for 74% of exposed women, was evaluated to assess whether biological susceptibility was related to timing of exposure. Although gestational age at first exposure ranged considerably within the cohorts, mean values were generally lower (i.e., earlier) in the Horne cohort. In cross-tabulations of dose and timing, the data were dichotomized at 13 weeks gestational age because the first trimester represents a period of heightened susceptibility for the developing fetal reproductive system (Sadler 2004).

Information on DES dose and timing was available for 33% of exposed women: 100% of Dieckmann participants, 30% of DESAD, 70% of Horne, and 0% of WHS. Women with complete data on dose and timing were more likely than those without complete data to be younger (year of birth 1960 or later: 18.2% vs. 11.5%), report a history of infertility (41.3% vs. 33.3%), and be primiparous (34.5% vs. 27.9%). No significant differences were found with respect to maternal age at first birth, age at menarche, education, body mass index (BMI), or smoking status.

On both the 1994 and 1997 questionnaires, women reported the outcome of each pregnancy (singleton vs. multiple live birth, stillbirth, spontaneous abortion, induced abortion, and other), the sex of each birth, and the date of the child’s birth. Infertility history was elicited on the 1994 questionnaire using two commonly employed definitions: whether the participant ever tried to become pregnant for ≥ 12 months without success, and whether the participant ever sought medical assistance for infertility from a health care provider. Women were also asked if they had ever used fertility drugs.

### Statistical methods

Participants were allowed to contribute more than one birth to the analysis. Analyses were restricted to 7,732 singleton live births with complete information on offspring sex and birth date (4,968 exposed and 2,764 unexposed). We estimated odds ratios (ORs) and 95% confidence intervals (CIs) for the association of prenatal DES exposure with secondary sex ratio using generalized estimating equations (GEE) to account for nonindependence (Liang and Zeger 1993). ORs overestimate prevalence ratios when the outcome is common, as was the case in our study. Nevertheless, we present ORs for comparability with other studies of secondary sex ratio.

We considered maternal age, cigarette smoking, calendar year of child’s birth, parity (i.e., birth order), and the use of fertility drugs as potential confounders. Factors associated with DES exposure that changed the OR by > 2% were included in the final regression models. Based on these criteria, multivariable models controlled for maternal age at conception (< 25, 25–29, 30–34, 35–39, ≥ 40 years), parity (1, 2, ≥ 3), and calendar year of child’s birth (before 1980, 1980–1984–1985–1989–1990 or later). Models were further adjusted for cohort (DESAD, Dieckmann, Horne, WHS) because of cohort-related differences in study methodology and participant recruitment. Tests for trend by dose or timing were performed by adding to the regression model a single ordinal term coded as 0, 1, and 2 for no DES, low dose, and high dose, or 0, 1, 2, and 3 for no DES, first exposure at 13 weeks or later, 9–12 weeks, and before 9 weeks, respectively (Breslow and Day 1987). Because plasma levels of endogenous estrogens have been shown to decrease with increasing age (Dorgan et al. 1995) and parity (Bernstein et al. 1986), we evaluated whether maternal age (< 30 vs. ≥ 30 years) and parity (1 vs. ≥ 2 live births) modified the association between DES and sex ratio. We also examined interaction by cohort. Likelihood ratio tests were used to evaluate statistical interaction by comparing models with and without cross-product terms between DES exposure and covariates of interest.

Finally, it is well established that *in utero* DES exposure is associated with decreased fertility (Kaufman et al. 2000; Palmer et al. 2001). Although GEE analyses that include multiple births per mother provide additional statistical power, the use of all births could conceivably bias results toward the null if the influence of DES varies among exposed women. For example, if there are women who are less sensitive to the effects of DES, and the effect of DES on parity is related to its effect on sex ratio, then the GEE approach may overrepresent DES-exposed women who are less sensitive to its effects (e.g., multiparous women). To address this concern, we performed a separate logistic regression analysis among first-borns only (i.e., one birth per mother), which may represent a less biased sample. Analyses were carried out using SAS statistical software (SAS Institute Inc. 2004).

## Results

Women exposed to DES *in utero* were slightly younger, more educated, more likely to report a history of infertility and use fertility drugs, and less likely to have ever smoked cigarettes than unexposed women (Table 1). Exposed and unexposed women were similar with respect to BMI at 20 years of age, ever use of oral contraceptives, sexual history, and alcohol consumption. Over 96% of women were white (data not shown).

Table 2 shows the association between prenatal DES exposure and secondary sex ratio, overall and according to DES dose and gestational age at first exposure. DES-exposed women gave birth to 2,607 boys and 2,361 girls (sex ratio = 0.525), and unexposed women gave birth to 1,406 boys and 1,358 girls (sex ratio = 0.509), resulting in an unadjusted OR for having a male birth of 1.07 (95% CI, 0.97–1.17). After adjustment for maternal age, calendar year of child’s birth, parity, and study cohort, the OR for having a male infant comparing exposed to unexposed women was 1.05 (95% CI, 0.95–1.17).

The odds of conceiving a male birth appeared to increase with increasing DES dose in both unadjusted and adjusted models (*p*-trend = 0.038). Among women with complete information on dose, the fully adjusted ORs for women prenatally exposed to < 5 g and to ≥ 5 g relative to unexposed women were 1.12 (95% CI, 0.95–1.33) and 1.16 (95% CI, 0.98–1.36), respectively. Given that only a small proportion of exposed women had dose information (36%), we repeated these analyses after assigning women with missing dose the median value of their study center. With dose imputation, the fully adjusted ORs were 1.05 (95% CI, 0.92–1.19) for < 5 g and 1.10 (95% CI, 0.97–1.26) for ≥ 5 g relative to no exposure (*p*-trend = 0.124).

The odds of conceiving a male birth increased slightly with decreasing gestational age at first DES exposure, but there was no statistical evidence of a trend (*p*-trend = 0.186). Among women with complete information on timing of DES exposure, the fully adjusted ORs for first exposure at ≥ 13, 9–12, and < 9 weeks of gestation relative to no exposure were 1.03 (95% CI, 0.89–1.18), 1.06 (95% CI, 0.92–1.23), and 1.08 (95% CI, 0.93–1.24), respectively.

Women first exposed to DES earlier in gestation and at higher doses had the highest odds of having a male birth (Table 2). Among women with complete information on DES dose and timing (33%), the fully adjusted ORs for having a male birth were 0.91 (95% CI, 0.65–1.27) for first exposure at ≥ 13 weeks to < 5 g, 0.95 (95% CI, 0.71–1.27) for first exposure at ≥ 13 weeks to ≥ 5 g, 1.16 (95% CI, 0.96–1.41) for first exposure at < 13 weeks to < 5 g, and 1.24 (95% CI, 1.04–1.48) for first exposure at < 13 weeks to ≥ 5 g, compared with no exposure. In analyses that imputed doses for women with missing data, results were attenuated but generally similar. The fully adjusted ORs for having a male birth were 1.03 (95% CI, 0.86–1.22) for first exposure at ≥ 13 weeks to < 5 g, 1.03 (95% CI, 0.84–1.23) for first exposure at ≥ 13 weeks to ≥ 5 g, 1.02 (95% CI, 0.88–1.18) for first exposure at < 13 weeks to < 5 g, and 1.13 (95% CI, 0.97–1.31) for first exposure at < 13 weeks to ≥ 5 g compared with no exposure.

Although the association between DES and sex ratio appeared to be stronger for participants in the Horne cohort (Table 3), the association was based on small numbers and was not statistically different from the other cohorts. Dose and timing results among the DESAD cohort were consistent with those found in the overall sample. Dose and timing results could not be assessed in the WHS, Dieckmann, and Horne cohorts either because of lack of data (i.e., WHS cohort) or because of limited variation in dose (i.e., all of the Dieckmann participants received > 5 g) or timing (i.e., all Horne participants were first exposed to DES before 9 weeks of gestation).

The adjusted OR for the association between DES and sex ratio was similar among women with (OR = 1.03; 95% CI, 0.83–1.29) and without (OR = 1.03; 95% CI, 0.92–1.17) a history of infertility, defined as women who tried for ≥ 12 months to conceive without success or sought medical assistance for infertility (*p*-interaction = 0.975). Within the subgroup of women with a history of infertility, the OR was 1.00 among women who had used fertility drugs (95% CI, 0.68–1.47) and 1.05 among women who had not (95% CI, 0.80–1.37; *p*-interaction = 0.744). Likewise, the association between DES and sex ratio was similar among women < 30 years of age at the time of delivery (OR = 1.05; 95% CI, 0.91–1.20) compared with those ≥ 30 years of age (OR = 1.06; 95% CI, 0.91–1.24; *p*-interaction = 0.952). With respect to parity status at the time of birth, the adjusted OR was not significantly different among primiparous women (OR = 1.10; 95% CI, 0.95–1.28) compared with multiparous women (OR = 1.02; 95% CI, 0.88–1.17; *p*-interaction = 0.317). OR estimates for dose and timing were similar across these subgroups and showed no evidence of statistical interaction (data not shown).

In analyses restricted to first births only, overall and dose-specific results were generally stronger than results among all births (Table 4). The fully adjusted OR for having a male birth was 1.37 (95% CI, 1.06–1.77) for first exposure at < 13 weeks to ≥ 5 g compared with no exposure.

## Discussion

The present findings are based on the largest study to date of U.S. women with documented intrauterine exposure to DES. Although we found no overall association between *in utero* DES exposure and secondary sex ratio, DES-exposed women who were first exposed earlier in gestation and to higher doses gave birth to a significantly higher proportion of males. If the developing female reproductive system is more susceptible to endocrine disruptors in the first trimester, the stronger association observed for women exposed to higher DES doses earlier in gestation is biologically plausible (Sadler 2004). These findings are the first to suggest a link between *in utero* DES exposure among women and the sex ratio of their offspring.

Previous research on maternal exposure to endocrine disruptors and secondary sex ratio has focused on exposure at times other than the prenatal period. Although our finding of an increased sex ratio among DES-exposed women is not consistent with two positive studies that found a significant decrease in sex ratio after maternal exposure to polychlorinated biphenyls (PCBs) (Weisskopf et al. 2003) and mercury (Sakamoto et al. 2001), it is consistent with a small study of preconception maternal PCB concentrations (Taylor et al. 2006). In the latter study, the odds of a male birth were elevated among women in the second (OR = 1.29) and third (OR = 1.48) tertiles of estrogenic PCBs, whereas odds were reduced among women in the highest tertile (OR = 0.70) of antiestrogenic PCBs (Taylor et al. 2006). Although the sample size was small (*n* = 99) and the results were not statistically significant, the Taylor et al. (2006) study suggests that maternal exposure to chemicals with estrogenic properties might increase the likelihood of a male birth.

An important limitation of the present study is the high proportion of missing data on dose and timing of DES exposure (67% of exposed). A significant finding was observed only among those exposed earlier in gestation and to higher doses, but it is unclear whether or how our results would have changed had we acquired complete data on dose and timing. Among the exposed participants, the male birth proportions for women who did (0.543) and did not (0.516) have complete data on dose and timing were both higher than the unexposed (0.509), but the magnitude of the difference was noticeably higher in women with complete data on dose and timing. Although these differences cannot be downplayed, we believe it is unlikely that missingness of data was related to both DES dose (and timing) and the probability of a male birth—conditions that would be necessary for bias to occur. Analyses in which women with missing dose were assigned to the median dose of their field center produced attenuated ORs, as would be expected under random exposure misclassification, but the estimates were largely consistent with the main results. Moreover, when analyses were restricted to first births only—a sample that may be less biased because it is not over-represented by multiparous women—overall and dose-specific results were generally stronger. Nonetheless, our limited data on dose and timing should be taken into account when interpreting our results.

Strengths of the present study include the verification of DES exposure status by medical record and the determination of exposure status before reporting of birth outcomes, both of which reduce the potential for differential misclassification of exposure. It is unlikely that knowledge of one’s DES exposure influenced the reporting of offspring sex, as there is no information in the lay press about the influence of DES on secondary sex ratio. Given that similar proportions of exposed and unexposed women completed the 1994 questionnaire (87%), and no differences were found in the baseline characteristics of those who were and were not lost to follow-up (data not shown), selection bias is also an unlikely explanation of our results. Finally, in contrast to most studies of endocrine-disrupting compounds, we had a spectrum of data on dose and timing, which allowed for an examination of dose–response relations.

An association between *in utero* DES exposure and secondary sex ratio in women is biologically plausible. According to James (1987), environmental toxicants may influence secondary sex ratio via changes in maternal hormonal concentrations around the time of conception, with high concentrations of testosterone and estrogen increasing the probability of a son and high concentrations of gonadotropins and progesterone increasing the probability of a daughter. Another theory postulates that sex ratio is influenced by both oocyte maturation and the quality of cervical mucus (Jongbloet 2004). Given that both maturation and cervical liquefaction are influenced by estrogens before the midcycle, toxicants with antiestrogenic properties might be expected to increase the sex ratio.

Studies of prenatal DES exposure and its long-term effects on endogenous hormones are limited. The sole animal study of this relation showed that *in vitro* secretion of testosterone, total estrogen, and progesterone in ovarian tissue was significantly increased in female mice exposed prenatally to DES (Haney et al. 1984). However, tissue production *in vitro* may not necessarily reflect total secretion of these hormones *in vivo*, especially because the ovaries of exposed mice were smaller than those of unexposed mice. Only two human studies have examined differences in gonadotropins and sex hormones in association with *in utero* DES exposure (Peress et al. 1982; Wu et al. 1980), reporting that DES daughters had elevated levels of serum testosterone (Wu et al. 1980) but not estrogens (Peress et al. 1982; Wu et al. 1980), progesterone (Wu et al. 1980), or LH (Peress et al. 1982; Wu et al. 1980). Higher FSH levels among DES-exposed women were found only by Peress et al. (1982), but the FSH to LH ratio was unaffected. In the study by Wu et al. (1980), differences in testosterone were greatest in the postovulatory and perimenstrual phases of the menstrual cycle, suggesting that the corpus luteum of DES daughters produces more testosterone. Under James’ hormonal hypothesis (James 1987), elevated testosterone levels around the time of conception would be expected to increase the proportion of male births, as found in the present study. In contrast, the “over-ripeness ovopathy” hypothesis (Jongbloet 2004) seems less plausible given that no differences in plasma estrogen levels were observed between DES-exposed and unexposed women in either study (Peress et al. 1982; Wu et al. 1980).

The interpretation of the literature on secondary sex ratio has been subject to debate, particularly with respect to the veracity and significance of the declining sex ratios reported in several countries worldwide (Bonde and Wilcox 2007; Davis et al. 1998). Whereas some epidemiologists have argued for the use of sex ratio as a sentinel health indicator in response to environmental exposures (Davis et al. 1998), others have questioned its value, citing its vulnerability both to false positive reports and publication bias (Bonde and Wilcox 2007).

In conclusion, although we found no overall association between DES exposure *in utero* and secondary sex ratio, a small increase in the proportion of male births was observed among women who were first exposed earlier in gestation and to higher doses. These results warrant confirmation in other study populations with data on DES dose and timing. If the association is real, it adds to the growing concern held by some epidemiologists that endocrine disruptors may affect secondary sex ratio in humans.

## Figures and Tables

**Table 1:** Characteristics of parous women with and without prenatal DES exposure (1994).^a^

	Prenatal exposure to DES	
Characteristic	Yes (*n =* 2,496)	No (*n =* 1,275)
Cohort		
DESAD	2,090 (83.7)	607 (47.6)
Dieckmann	137 (5.5)	156 (12.2)
Horne	70 (2.8)	46 (3.6)
WHS	199 (8.0)	466 (36.6)
Year of birth		
Before 1950	463 (18.5)	374 (29.3)
1950–1954	1,063 (42.6)	537 (42.1)
1955–1959	629 (25.2)	300 (23.6)
1960 or later	341 (13.7)	64 (5.0)
Education		
High school or less	393 (15.8)	292 (22.9)
Some college	644 (25.9)	353 (27.7)
College	840 (33.7)	374 (29.4)
Graduate school	612 (24.6)	254 (20.0)
Age at menarche (years)		
< 12	394 (15.8)	223 (17.5)
12–13	1,510 (60.6)	737 (57.9)
≥ 14	589 (23.6)	313 (24.6)
BMI at 20 years of age (kg/m^2^)		
< 20	1,051 (43.4)	522 (42.3)
20–24	1,200 (49.6)	626 (50.7)
≥ 25	169 (7.0)	86 (7.0)
Use of fertility drugs		
Never	2,108 (84.7)	1,185 (93.0)
Ever	382 (15.3)	89 (7.0)
Smoking status		
Never	1,442 (58.0)	640 (50.3)
Ever	1,046 (42.0)	632 (49.7)
No. of live births		
1	751 (30.1)	283 (22.2)
2	1,191 (47.7)	631 (49.5)
≥ 3	554 (22.2)	361 (28.3)
Infertility history		
No	1,583 (64.0)	1,030 (81.5)
Yes^b^	889 (36.0)	234 (18.5)

a Values are expressed as number (column percent); numbers may not sum to total because of missing data.

b Defined as having tried to conceive for ≥ 12 months without success or having sought medical help for infertility.

**Table 2:** Offspring sex ratio in relation to prenatal DES exposure, overall and by timing and dose.

	No. of children					
	Male	Female	Proportion male	Unadjusted OR (95% CI)	Adjusted OR (95% CI)^a^	*p*-Value, test for trend^a^
Unexposed	1,406	1,358	0.509	1.00^b^	1.00^b^	—
Exposed	2,607	2,361	0.525	1.07 (0.97–1.17)	1.05 (0.95–1.17)	
DES dose (g)						
< 5	444	378	0.540	1.13 (0.96–1.33)	1.12 (0.95–1.33)	0.038
≥ 5	494	405	0.550	1.18 (1.01–1.37)	1.16 (0.98–1.36)	
Gestational age at first exposure (weeks)						
≥ 13	683	632	0.519	1.05 (0.91–1.19)	1.03 (0.89–1.18)	0.186
9–12	584	516	0.531	1.09 (0.95–1.26)	1.06 (0.92–1.23)	
< 9	687	609	0.530	1.09 (0.95–1.25)	1.08 (0.93–1.24)	
Dose and timing						
< 5 g, ≥ 13 weeks	98	101	0.493	0.94 (0.67–1.30)	0.91 (0.65–1.27)	—
≥ 5 g, ≥ 13 weeks	103	102	0.502	0.98 (0.74–1.30)	0.95 (0.71–1.27)	
< 5 g, < 13 weeks	293	241	0.549	1.17 (0.97–1.41)	1.16 (0.96–1.41)	
≥ 5 g, < 13 weeks	369	282	0.567	1.26 (1.07–1.50)	1.24 (1.04–1.48)	

Data on dose, timing, and both dose and timing were available for 36%, 74%, and 33% of women, respectively.

a Adjusted for maternal age at conception, year of child’s birth, parity, and cohort.

b Reference group for all column comparisons.

**Table 3:** Offspring sex ratio in relation to prenatal DES exposure by study cohort.

	No. of children					
Cohort	Male	Female	Proportion male	Unadjusted OR (95% CI)	Adjusted OR (95% CI)^a^	*p*-Value, test for interaction
DESAD						
Unexposed	671	639	0.512	1.00^b^	1.00^b^	—b
Exposed	2,204	1,990	0.526	1.05 (0.93–1.19)	1.06 (0.94–1.20)	
Dieckmann						
Unexposed	179	162	0.525	1.00^b^	1.00^b^	0.85
Exposed	146	121	0.547	1.10 (0.80–1.53)	1.08 (0.78–1.51)	
Horne						
Unexposed	34	48	0.415	1.00^b^	1.00^b^	0.14
Exposed	57	48	0.543	1.67 (0.94–2.99)	1.61 (0.86–3.02)	
WHS						
Unexposed	522	509	0.506	1.00^b^	1.00^b^	0.51
Exposed	200	202	0.498	0.97 (0.76–1.23)	0.95 (0.75–1.20)	

a Adjusted for maternal age at conception, year of child’s birth, and parity.

b Reference group for interaction tests.

**Table 4:** Offspring sex ratio in relation to prenatal DES exposure, overall and by timing and dose, restricted to first births.

	No. of children					
	Male	Female	Proportion male	Unadjusted OR (95% CI)	Adjusted OR (95% CI)^a^	*p*-Value, test for trend^a^
Unexposed	650	625	0.510	1.00^b^	1.00^b^	—
Exposed	1,339	1,155	0.537	1.12 (0.97–1.28)	1.10 (0.95–1.28)	
DES dose (g)						
< 5	229	197	0.538	1.12 (0.90–1.39)	1.11 (0.88–1.40)	0.021
≥ 5	261	202	0.564	1.24 (1.00–1.54)	1.25 (1.00–1.57)	
Gestational age at first exposure (weeks)						
≥ 13	347	284	0.550	1.17 (0.97–1.42)	1.17 (0.95–1.44)	0.098
9–12	288	265	0.521	1.05 (0.86–1.28)	1.03 (0.83–1.28)	
< 9	377	305	0.553	1.19 (0.99–1.43)	1.20 (0.98–1.47)	
Dose and timing						
< 5 g, ≥ 13 weeks	54	49	0.524	1.06 (0.71–1.58)	1.03 (0.68–1.55)	—
≥ 5 g, ≥ 13 weeks	48	50	0.490	0.92 (0.61–1.39)	0.92 (0.61–1.40)	
< 5 g, < 13 weeks	147	130	0.531	1.09 (0.84–1.41)	1.09 (0.83–1.43)	
≥ 5 g, < 13 weeks	200	142	0.585	1.35 (1.06–1.72)	1.37 (1.06–1.77)	

Data on dose, timing, and both dose and timing were available for 36%, 75%, and 33% of women, respectively.

a Adjusted for maternal age at conception, year of child’s birth, and cohort.

b Reference group for all column comparisons.
